# Debridement, antimikrobielle Therapie und Implantaterhalt bei akuten periprothetischen Infektionen

**DOI:** 10.1007/s00132-025-04716-6

**Published:** 2025-09-06

**Authors:** Dirk Müller, Stephan Kirschner, Benjamin Schloßmacher, Rüdiger von Eisenhart-Rothe, Igor Lazic

**Affiliations:** 1 Technische Universität München, Klinikum rechts der Isar, Klinik und Poliklinik für Orthopädie und Sportorthopädie, Ismaninger Str. 22, 81675 München, Deutschland; 2 Klinik für Orthopädie, ViDia-Kliniken, Steinhäuserstr. 18, 76135 Karlsruhe, Deutschland

**Keywords:** Periprothetische Infektion (PPI), DAIR-Verfahren, Implantaterhalt, Antibiotikatherapie, *Staphylococcus aureus*, Periprosthetic joint infection (PJI), DAIR procedure, Implant retention, Antimicrobial therapy, *Staphylococcus aureus*

## Abstract

**Hintergrund:**

Das DAIR-Verfahren (Debridement, Antibiotikatherapie und Implantaterhalt) stellt eine Behandlungsoption für akute periprothetische Infektionen (PPI) dar. Im Gegensatz zur Revisionsendoprothetik ist es technisch weniger invasiv und kann bei geeigneter Indikation den Erhalt einer fest verankerten Endoprothese ermöglichen. Der Therapieerfolg hängt jedoch von einer Vielzahl patienten-, keim- und operationsbezogener Faktoren ab.

**Methoden:**

Basierend auf einer umfassenden Literaturrecherche aktueller internationaler Studien und Leitlinien wurden die entscheidenden Einflussgrößen für den Erfolg eines DAIR-Verfahrens systematisch dargestellt. Zusätzlich wurden klinische Erfahrungen aus der eigenen orthopädischen Klinik, insbesondere bei Infektionen mit Staphylokokken, ausgewertet und eingebunden.

**Ergebnisse:**

Die Analyse identifizierte entscheidende Erfolgsfaktoren für das DAIR-Verfahren: frühzeitige Intervention innerhalb von 3 Wochen nach Symptombeginn, Austausch aller mobilen Komponenten, stabiles Implantat, intakter Weichteilmantel sowie die Berücksichtigung von Risikofaktoren wie hohes Alter, immunsuppressive Therapie, COPD oder rheumatoide Arthritis. Die Wahl des antibiotischen Regimes, insbesondere die Kombination mit Rifampicin bei Staphylokokken-Infektionen, beeinflusst das Outcome wesentlich.

**Schlussfolgerung:**

DAIR kann unter definierten Voraussetzungen eine effektive Therapieoption mit guten Heilungschancen darstellen. Eine sorgfältige Patientenselektion, multidisziplinäre Fallbesprechung und konsequente Umsetzung chirurgischer und antiinfektiver Standards sind essenziell, um die Erfolgsrate zu verbessern. Bei ungünstiger Ausgangslage sollte jedoch ein direkter Prothesenwechsel in Erwägung gezogen werden.

## Abstract (en)

**Background:**

The DAIR procedure (debridement, antibiotic therapy, and implant retention) represents a treatment option for acute periprosthetic joint infections (PJI). Compared to revision arthroplasty, it is technically less invasive and, under appropriate indications, can preserve a well-fixed endoprosthesis. However, treatment success depends on numerous patient-, pathogen-, and procedure-related factors.

**Methods:**

Based on a comprehensive review of current international studies and guidelines, we systematically analyzed the key factors influencing the success of DAIR. In addition, clinical experiences from our orthopedic department, particularly with staphylococcal infections, were evaluated and integrated.

**Results:**

Critical success factors for DAIR include early intervention within 3 weeks of symptom onset, exchange of all mobile components, stable implant fixation, intact soft tissue envelope, and consideration of risk factors such as advanced age, immunosuppressive therapy, COPD, or rheumatoid arthritis. The choice of antibiotic regimen, especially rifampicin-based combination therapy for staphylococcal infections, affects the outcome significantly.

**Conclusion:**

DAIR can be an effective treatment option with favorable healing rates under clearly defined conditions. Careful patient selection, a multidisciplinary case review, and strict adherence to surgical and antimicrobial standards are essential to improve success rates. In unfavorable clinical situations, a direct implant revision should be considered instead.

## Hintergrund

Der endoprothetische Gelenkersatz hat die Behandlung von Patient:innen mit Arthrose revolutioniert, da er eine langfristige Schmerzlinderung und die Wiederherstellung der Mobilität ermöglicht. Die wohl gefürchtetste Komplikation nach einem endoprothetischen Gelenkersatz ist die periprothetische Gelenkinfektion (PPI). Trotz Fortschritten in der Prävention, liegt die Inzidenz einer PPI nach primärem Gelenkersatz zwischen 1 und 2 % [14, 15], nach Revisionsoperationen steigt diese Rate auf etwa 4 % an [22].

Kurative chirurgische Behandlungsstrategien können entweder prothesenerhaltend durchgeführt werden oder es erfolgt der Prothesenwechsel. Das prothesenerhaltende Vorgehen wird im Englischen als „DAIR“ bezeichnet. Der Begrifft steht hierbei für „debridement, antibiotics and implant retention“.

In diesem Beitrag analysieren wir die wichtigsten Einflussfaktoren auf den Therapieerfolg des DAIR-Verfahrens auf Basis der aktuellen wissenschaftlichen Literatur. Wir definieren klare Indikationen und Kontraindikationen für die Durchführung eines DAIR-Eingriffs auf Basis der Empfehlungen der European Bone and Joint Infection Society (EBJIS) [31] und bieten eine praxisorientierte Anleitung zur operativen Technik sowie zur antimikrobiellen Therapie im Rahmen des DAIR-Managements. Darüber hinaus präsentieren wir die klinischen Ergebnisse unserer Einrichtung mit dem DAIR-Verfahren – mit besonderem Fokus auf PPI verursacht durch Staphylokokken.

## Indikationen, Kontraindikationen und Risikofaktoren für ein DAIR-Verfahren

Beim DAIR-Verfahren ist es das Ziel, die Entfernung einer fest verankerten Prothese vermeiden. Die Versagensrate nach DAIR variiert stark und liegt in neueren Publikationen zwischen 7 und 55 % [6, 7, 11, 24, 25, 29, 35, 37, 39, 42, 43].

**Abb. 1:**
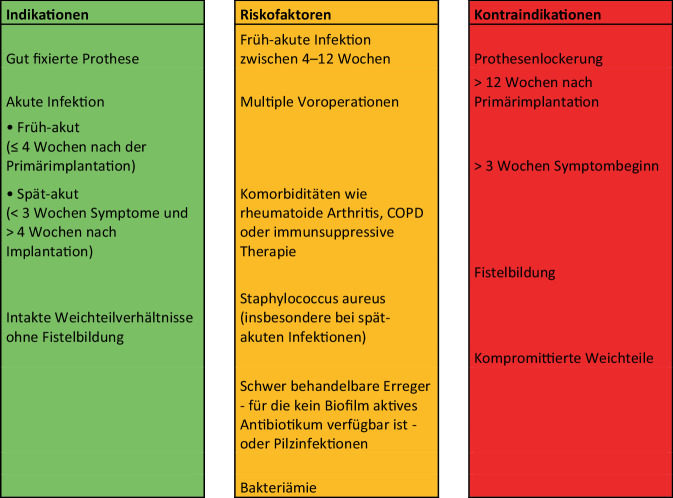
Indikationen, zu berücksichtigende Risikofaktoren und Kontraindikationen für ein DAIR (Debridement, Antibiotikatherapie und Implantaterhalt)-Verfahren gemäß aktueller Literatur. *COPD* „Chronic obstructive pulmonary disease“ (Adaptiert nach: Sigmund et al. 2025 [31], Journal of Bone and Joint Infection, © Autor:innen 2025. Veröffentlicht unter der Creative Commons Lizenz CC BY 4.0. 10.5194/jbji-10-101-2025)

Die Indikation für ein DAIR-Verfahren wird von verschiedenen Autor:innen und Fachgesellschaften unterschiedlich definiert [1, 23]. In Abb. 1 sind die Indikationen und Kontraindikationen, sowie die Risikofaktoren für das Scheitern eines DAIR-Verfahrens nach den aktuellen Empfehlungen der EBJIS zusammengefasst [31].

### Biofilmbildung und Infektionstyp

Periprothetische Infektionen werden in akute und chronische Infektionen unterteilt, dies liegt in der Fähigkeit der Mikroorganismen zur Biofilmbildung auf Implantatoberflächen. Sobald der Biofilm vollständig ausgereift ist, wird ein vollständiger Prothesenwechsel erforderlich. Bei akuten PPI wird angenommen, dass der Biofilm noch nicht vollständig ausgereift ist.

Akute PPI werden weiter in früh- und spätakute Infektionen unterteilt. Die frühakute Infektion beschreibt die postoperative Infektion. Die spätakute Infektion entsteht durch eine hämatogene Einstreuung in das Gelenk. Gemäß den aktuellen Empfehlungen der EBJIS tritt eine frühakute PPI in den ersten 4 Wochen nach der Indexoperation auf. Eine spätakute PPI liegt vor, wenn Patient:innen nach einem komplikationslosen postoperativen Verlauf > 4 Wochen plötzlich Symptome entwickeln, die < 3 Wochen andauern [31]. In Studien wurde eine signifikant geringere Erfolgsrate bei spätakuten im Vergleich zu frühakuten PPI festgestellt [41]. Die höhere Misserfolgsrate kann möglicherweise durch eine ungenaue Bestimmung des Symptombeginns erklärt werden.

**Abb. 2:**
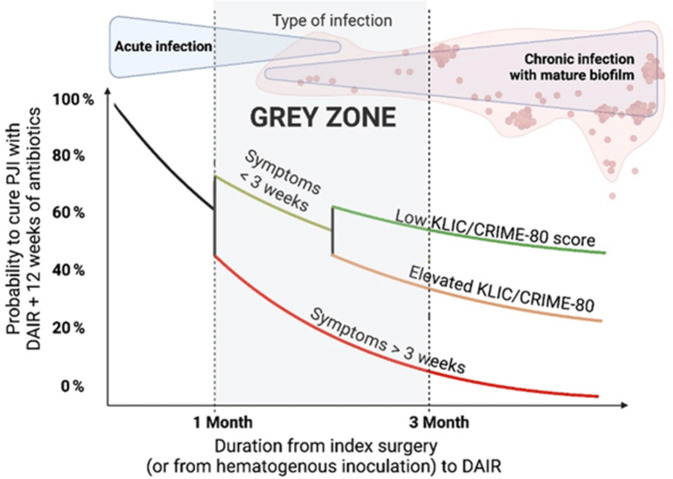
Wahrscheinlichkeit der Heilung einer periprothetischen Gelenkinfektion (*PJI*) in Abhängigkeit von der Dauer der Symptome. *DAIR* Debridement, Antibiotikatherapie und Implantaterhalt, *KLIC score* Risikobewertungsscore für frühakute periprothetische Infektion [38], *CRIME-80 score* Risikobewertungsscore für spätakute periprothetische Infektion [42]. (Abbildung übernommen aus: Sigmund et al. 2025 [31], Journal of Bone and Joint Infection, © Autor:innen 2025. Veröffentlicht unter der Creative Commons Lizenz CC BY 4.0. 10.5194/jbji-10-101-2025)

Mit zunehmender Symptomdauer nimmt die Erfolgswahrscheinlichkeit eines DAIR-Verfahrens ab [1, 23, 28, 39]. In den aktuellen Empfehlungen der EBJIS [31] wird empfohlen, bei akuter PPI zeitnah ein DAIR-Verfahren durchzuführen. Es wird empfohlen, ein DAIR-Verfahren innerhalb von 3 Wochen nach Symptombeginn durchzuführen – vorzugsweise innerhalb der ersten 7 Tage (Abb. 2).

### Art der Endoprothese

In einigen Studien wurden höhere Erfolgsraten bei Hüftprothesen im Vergleich zu Knieprothesen berichtet [12, 17], andere Studien zeigten jedoch keinen signifikanten Unterschied. Ein DAIR-Verfahren kann daher sowohl bei Hüft- als auch bei Knieendoprothesen durchgeführt werden. Die Misserfolgsraten von DAIR-Verfahren nach Revisionsendoprothetik und insbesondere Megaprothesen sind erhöht, für infizierte Megaprothesen wurden Erfolgsraten von 50 % beschrieben [2].

### Patientenbezogene Risikofaktoren

Verschiedene patientenbezogene Faktoren für das Versagen eines DAIR-Verfahrens sind bekannt:

- *Alter: *Über 80 Jahre bedeutet höheres Risiko [42].
- *Rheumatoide Arthritis: *Erhöhtes Risiko [19, 29, 42].
- *Immunsuppressive Therapie:* Erhöhtes Risiko [19, 29, 42].
- *Adipositas und Body-Mass-Index (BMI): *Erhöhtes Infektionsrisiko nach primärer Endoprothetik [10], Risiko für DAIR-Verfahren unklar.
- *Rauchen und chronisch obstruktive Lungenerkrankung (COPD): *Rauchen keine signifikante Korrelation mit Versagen eines DAIR-Verfahrens [21, 29, 39, 43], COPD korreliert mit Therapieversagen eines DAIR [29].
- *C‑reaktives Protein (CRP): *Präoperativer CRP-Wert > 11,5 mg/dl ist Prädiktor für Therapieversagen des DAIR [5, 29, 38, 42, 43].
- *KLIC-Score: *Risikobewertungsscore für frühakute PPI. Berücksichtigt 5 präoperative Faktoren: chronische Niereninsuffizienz, Leberzirrhose, Revisionsoperation oder Schenkelhalsfraktur, CRP > 11,5 mg/dl, zementierte Prothese [38].
- *CRIME-80-Score: *Risikobewertungsscore für spätakute PPI. Berücksichtigte Risikofaktoren für ein Therapieversagen: COPD, CRP > 150 mg/l, rheumatoide Arthritis, Indikation zur Prothesenimplantation (z. B. Fraktur), männliches Geschlecht, Nichtmöglichkeit des Austauschs mobiler Komponenten, Alter über 80 Jahre [42].

### Mikroorganismen

Der verursachende Mikroorganismus hat entscheidenden Einfluss auf den Erfolg eines DAIR-Verfahrens:

- *Staphylococcus aureus (STAU): *Erhöhtes Risiko für das Versagen einer DAIR-Therapie (Therapieversagens 25–56 %) [5, 29, 42, 43]. 11 % höhere Versagensrate im Vergleich zu anderen Erregern [29].
- *Koagulase-negative Staphylokokken (CoNS*): Therapieversagen 27–44 % [35, 44].
- *Streptokokken*: Therapieversagen 9–52 % [13, 43].
- *Enterokokken*: Therapieversagen 8–73 % [42, 44].
- *Gramnegative Bakterien*: Therapieversagen 25–56 % [13, 44].
- *Polymikrobielle Infektionen: *Erhöhtes Risiko für das Versagen einer DAIR-Therapie [5, 6, 29].
- *Kulturnegative Infektionen*: Kulturnegative Infektionen zeigen nach einem DAIR-Verfahren tendenziell bessere Ergebnisse im Vergleich zu kulturpositiven Fällen [6, 36].
- *Bakteriämie/Sepsis*: Das Vorliegen einer Bakteriämie gilt als wichtiger Risikofaktor für das Versagen eines DAIR-Verfahrens [20, 42]. Aufgrund der hohen Misserfolgsrate sollte bei Bakteriämie oder Sepsis eine zweizeitiger Prothesenwechsel erfolgen.

## Chirurgisches Vorgehen

Beim DAIR-Verfahren ist ein offenes Vorgehen anzustreben. In Studien konnten höhere Erfolgsrate bei einem offenen Débridement im Vergleich zu einem arthroskopischen Auswaschen gezeigt werden [16]. Zudem ist der Austausch der mobilen Komponenten im Rahmen eines DAIR-Verfahrens mit besseren Ergebnissen assoziiert [5, 19, 29, 37, 42, 43], was nur bei einem offenen Vorgehen möglich ist.

Bei DAIR-Eingriffen sollte nach Möglichkeit der vorherige Hautschnitt verwendet werden. Zur optimalen Darstellung und Exposition des Gelenks kann eine Erweiterung der Inzision notwendig sein. In Fällen mit nekrotischer oder hypertropher Narbe sollten die Wundränder exzidiert werden. Nach Eröffnung des Gelenks ist eine standardisierte Gewebeentnahme unter streng aseptischen Bedingungen essenziell. Es werden 4–6 Gewebeproben für mikrobiologische Untersuchungen empfohlen, um zuverlässige Ergebnisse zu gewährleisten [8]. Für die histopathologische Diagnostik sollte mindestens eine Probe entnommen werden. Neuere Studien empfehlen sogar 3–6 Gewebeproben aus infektverdächtigem Areal zur histopathologischen Aufarbeitung zu entnehmen, um die diagnostische Güte insbesondere bei chronischen Infektionen zu erhöhen [32].

Der nächste Schritt ist das chirurgische Débridement purulenter Ansammlungen und periartikulären Gewebes. Alle makroskopisch infizierten, nicht vitalen und kontaminierten Gewebeanteile werden sorgfältig entfernt, eine Synovektomie wird durchgeführt. Im englischsprachigen Raum wird auch von „onkologischer“ Resektion gesprochen, um die Radikalität der Synovektomie zu beschreiben. Beim DAIR-Verfahren ist es jedoch wichtig, vitale Gewebe und Schlüsselstrukturen möglichst zu erhalten, um eine Gelenkinstabilität zu vermeiden – gleichzeitig müssen alle potenziell infizierten oder nekrotischen Gewebeanteile konsequent exzidiert werden. Die mobilen Komponenten werden entfernt (z. B. Polyethylen bei Knieprothesen, Inlay und Kopf bei Hüftprothesen). Anschließend wird die Stabilität und Osseointegration der verbleibenden Prothese geprüft. Ist die Prothese gelockert oder nicht gut integriert, wird eine vollständige Prothesenexplantation empfohlen. Nur bei stabil verankerten Prothesen sollte das DAIR-Verfahren fortgeführt werden. Nach der Entfernung der mobilen Komponenten erfolgt ein weiteres gründliches Débridement zuvor nicht erreichbarer Bereiche.

Zur Reduktion der bakteriellen Last wird eine reichliche Spülung von Gewebe, Knochen und der Oberfläche der verbleibenden Prothese mit 0,9 %iger NaCl-Lösung durchgeführt. Es liegen keine klinischen Daten zur optimalen Spülmenge vor. Die meisten Zentren verwenden zwischen 3 und 9 l Kochsalzlösung [5, 6, 37, 40, 43]. Bezüglich der optimalen Spüllösung führten Siddiqi et al. eine Übersichtsarbeit durch, in der sie Spüllösungen zur Behandlung von PPI untersuchten. Antiseptika – darunter Povidon-Iod, Chlorhexidinglukonat, Wasserstoffperoxid sowie vorformulierte Kombinationslösungen – können erfolgreich in der PPI-Behandlung eingesetzt werden [30].

Nach ausgiebiger Lavage werden neue mobile Komponenten (z. B. Inlay, Kopf) eingesetzt und fixiert. Falls keine neuen Komponenten verfügbar sind, sollten die entfernten Teile gründlich gereinigt, in eine Antiseptikalösung eingelegt und anschließend wieder implantiert werden. Dabei muss jedoch davon ausgegangen werden, dass diese Komponenten weiterhin eine Keimlast tragen (Tab. 1).

**Tab. 1:** Operationsschritte der DAIR-Prozedur (Debridement, Antibiotikatherapie und Implantaterhalt)

Operative Technik	
1.	Offene Arthrotomie
2.	Standardisierte Gewinnung tiefer Gewebeproben (1 Aspiration, 4–6 Gewebeproben)
3.	Adäquates und standardisiertes chirurgisches Débridement
4.	Entfernung der mobilen Komponenten
5.	Überprüfung der Prothesenstabilität (fest sitzend)
6.	Zweites gründliches Débridement
7.	Spülung
8.	Einsetzen neuer mobiler Komponenten

## Systemische antibiotische Therapie

Die Wahl der richtigen empirischen antibiotischen Therapie nach chirurgischem Débridement, welche die häufigsten grampositiven PPI-Erreger abdeckt, ist entscheidend für den Erfolg einer DAIR-Behandlung. Dabei ist es entscheidend, die lokale Epidemiologie und Resistenzmuster zu kennen, um die geeignete antimikrobielle Therapie nach DAIR zu beginnen. Bei Patient:innen ohne Sepsis kann die antibiotische Therapie bis zur intraoperativen Probenentnahme verzögert werden, um die Identifikation des Erregers zu erleichtern [33].

Um eine hohe intraartikuläre Antibiotikakonzentration zu gewährleisten, wird im deutschsprachigen Raum empfohlen, die Antibiotikatherapie zunächst 2 Wochen intravenös fortzuführen. Die OVIVA-Studie, durchgeführt bei verschiedenen Arten von Knochen- und Gelenkinfektionen, zeigte die Nichtunterlegenheit eines frühen Wechsels auf orale Antibiotika (z. B. innerhalb von 7 Tagen nach Operation) im Vergleich zu längerer i.v. Therapie [18]. Da diese Studie jedoch nicht exklusiv PPI betrachtete, sollte eine Verkürzung der intravenösen Therapie gerade beim DAIR-Verfahren kritisch hinterfragt werden.

Bei der Auswahl des oralen Antibiotikums ist auf eine gute orale Bioverfügbarkeit und Biofilmaktivität zu achten. Für Staphylokokken wird eine Kombinationstherapie aus Fluorchinolon und Rifampicin empfohlen [3, 9, 42]. Der Einsatz von Rifampicin bei anderen grampositiven Bakterien, wie Cutibacterium acnes oder Streptokokken, kann erwogen werden, ist aber nicht durch ausreichend Evidenz gedeckt. Bei gramnegativen Bakterien wird der Einsatz von Fluorchinolonen empfohlen [26]. Aufgrund erst neuerlicher Anwendungsbeschränkungen bei fluorchinolonhaltigen Antibiotika (Rote-Hand-Brief 2023), muss der Einsatz dieser Medikamente individuell nach Möglichkeit in Absprache mit Mikrobiolog:innen und Pharmazeut:innen entschieden werden.

Die Gesamtdauer der Antibiotikatherapie nach DAIR ist weiterhin umstritten. Die richtungsweisende DATIPO-Studie (2021) zeigte ein schlechteres Ergebnis bei 6‑wöchiger Therapie im Vergleich zu 12 Wochen [4]. Daher wird aktuell eine Gesamtdauer der Antibiotikatherapie von 12 Wochen empfohlen. Wir empfehlen, die Antibiotikatherapie nach Möglichkeit mit Infektiolog:innen oder Mikrobiolog:innen, die Erfahrung in der Behandlung implantatassoziierter Infektionen haben, individuell zu besprechen. Insbesondere aufgrund der Induktion des Zytochrom-P450-System durch Rifampicin bei insgesamt langen Therapiedauern ist es zudem sinnvoll, die medikamentöse Therapie mit Pharmazeut:innen abzustimmen (Tab. 2).

**Tab. 2:** Medizinische Empfehlungen zur DAIR-Prozedur (Debridement, Antibiotikatherapie und Implantaterhalt)

Medizinische Empfehlungen	
1.	Bei Fehlen einer Sepsis kann die Antibiotikatherapie bis nach der Gewebeentnahme aufgeschoben werden
2.	Eine Gesamtdauer von 12 Wochen Antibiotikatherapie, beginnend mit 2 Wochen intravenöser Initialphase, wird empfohlen
3.	Anpassung der kalkulierten Antibiotikatherapie nach Antibiogramm
4.	Bei Staphylokokken-bedingten Infektionen: Kombination aus Fluorchinolonen und Rifampicin; bei gramnegativen: Fluorchinolone (Cave: Rote-Hand-Brief 2023)

Die Applikation antimikrobieller Substanzen direkt an der Infektionsstelle kann zu höheren lokalen Konzentrationen führen. In den letzten 10 Jahren haben sich antibiotikabeladene Trägersysteme als vielversprechende Ergänzung zur Behandlung von PPI etabliert [34]. Während Knochenzementkügelchen aufgrund der Notwendigkeit der Entfernung nicht empfohlen werden können, kommen alternative Träger wie Calciumsulfat zunehmend zum Einsatz [34]. Aufgrund widersprüchlicher Ergebnisse in der aktuellen Literatur bleibt der Nutzen antibiotikabeladener Trägersysteme im Rahmen von DAIR-Verfahren derzeit unklar.

## Eigene Erfahrung mit dem DAIR-Verfahren

In einer eigenen Fallserie wurden PPI des Hüft- und Kniegelenkes mit Staphylokokken bei 177 Patient:innen nachuntersucht. In unserer Fallserie hatten 62 Patient:innen einen PPI mit *Staphylococcus aureus* (STAU) und 115 Patient:innen einen PPI mit Koagulase-negativen Staphylokokken (CoNS). Bei 55 Patient:innen erfolgte ein DAIR-Verfahren, bei 117 erfolgte ein Prothesenwechsel. Das mittlere Follow-Up betrug 30 Monate (Tab. 3).

**Tab. 3:** Patientendemografie und Daten zu periprothetischen Infektionen in unserer Kohorte

		Alle Patient:innen (*n* = 177)	*Staphylococcus aureus* (*n* = 62)	Koagulase-negative Staphylokokken (*n* = 115)
*Follow-Up in Monaten (Median; Interquartilsabstand)*		30 (51)	30 (46)	32 (51)
*Therapieverfahren *	DAIR (Debridement, Antibiotikatherapie und Implantaterhalt)	31,1 %	41,9 %	25,2 %
	Einzeitiger Prothesenwechsel	8,5 %	9,7 %	7,9 %
	Zweizeitiger Prothesenwechsel	44,1 %	30,7 %	51,3 %
	Mehrzeitiger Prothesenwechsel	13,5 %	14,5 %	13,0 %
	Lavage und Debridement, ohne Wechsel der mobilen Teile	2,8 %	3,2 %	2,6 %
*Prothesentyp *	Primärprothese	32,8 %	35,5 %	31,3 %
	Revisionsprothese	52,5 %	48,4 %	54,8 %
	Megaprothese	14,7 %	16,1 %	13,9 %

**Abb. 3:**
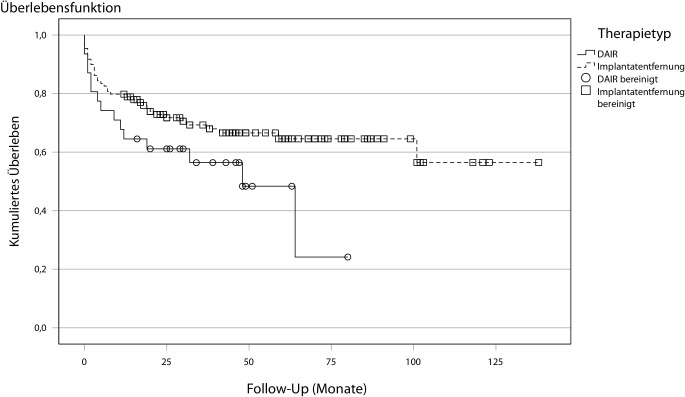
Infektfreies Überleben nach Debridement, Antibiotikatherapie und Implantaterhalt (*DAIR*) und Prothesenwechsel bei einer Symptomdauer >1 Woche (*n* = 140; *p* = 0,073)

Insgesamt zeigte sich bei einem DAIR-Verfahren eine hohe Versagensrate von 43,6 %. Bei einer Symptomdauer > 1 Woche zeigte sich ein höheres Therapieversagen des DAIR-Verfahrens gegenüber dem Prothesenwechsel, der Unterschied war jedoch statistisch nicht signifikant (*p* = 0,073; Abb. 3). Bei einer Symptomdauer über 4 Wochen zeigte sich eine signifikant höhere Versagensrate des DAIR-Verfahren gegenüber dem Prothesenwechsel (*p* = 0,004; Tab. 4).

**Tab. 4:** Therapieversagen von DAIR und Prothesenwechsel

	DAIR (*n* = 55) (%)	Prothesenwechsel (*n* = 117) (%)	*p*-Wert
Alle Patient:innen (*n* = 172)*	43,6	33,3	0,062
STAU (*n* = 60)	38,5	41,2	0,945
CoNS (*n* = 112)	48,3	30,1	0,040
Alle Patient:innen, Symptome < 4 Wochen (*n* = 63)	35,7	47,6	0,480
Alle Patient:innen, Symptome > 4 Wochen (*n* = 109)	69,2	30,2	0,004

*STAU* *Staphylococcus aureus*, *CoNS* Koagulase-negative Staphylokokken

*Patient:innen ohne Wechsel der mobilen Teile wurden ausgeschlossen (*n* = 5)

Die hohe Versagensrate des DAIR-Verfahrens bei Staphylokokken-bedingten PPI in unserer Fallserie steht im Einklang mit den bisherigen Literaturergebnissen. Auffällig ist jedoch die insgesamt hohe Misserfolgsrate sowohl bei DAIR als auch beim Prothesenwechsel. Diese könnte durch den hohen Anteil an Revisions- und Megaprothesen in unserer Kohorte erklärt werden. Insbesondere bei Megaprothesen ist aufgrund der größeren Implantatoberflächen von einem erhöhten Infektionsrisiko auszugehen.

Interessanterweise hatte das DAIR-Verfahren in unserer Fallserie bei Infektionen mit CoNS eine höhere Versagensrate als für STAU. Dies steht im Wiederspruch zu der verfügbaren Literatur, welche für STAU eine höhere Versagensrate berichtet als für CoNS. Da CoNS typische Keime für chronische PPI darstellen, vermuten wir, dass es sich bei einem Anteil der Fälle um akute Exazerbationen chronischer Infektionen handelt, die durch ein implantaterhaltendes Vorgehen nicht adäquat angegangen worden sind.

Unsere Fallserie unterstützt die Empfehlungen der EBJIS [31], ein DAIR-Verfahren vorzugsweise innerhalb der ersten 7 Tage nach Symptombeginn durchzuführen. Vier Wochen nach Symptombeginn sollte kein DAIR-Verfahren mit dem Ziel der Infekteradikation mehr durchgeführt werden. Basierend auf unserer Fallserie sollte die Indikation für ein DAIR-Verfahren sowohl bei PPI mit STAU als auch mit CoNS nur zurückhaltend gestellt werden.

## Schlussfolgerung

DAIR ist eine effektive Behandlungsoption mit guten Eradikationsraten bei sorgfältig ausgewählten Patient:innen. Durch die Vermeidung der Explantation einer in der Regel gut fixierten Prothese, können Patient:innen schneller in ihre Aktivitäten zurückkehren. Allerdings profitieren nicht alle Patient:innen von einer DAIR-Prozedur. Es wurde gezeigt, dass das Behandlungsergebnis von verschiedenen Faktoren abhängt und dass die Patientenauswahl entscheidend für den Erfolg von DAIR ist.

## Data Availability

Die in dieser Studie erhobenen Datensätze können auf begründete Anfrage beim Korrespondenzautor angefordert werden.

## Abkürzungen

BMI: Body-Mass-Index
CoNS: Koagulase-negative Staphylokokken
COPD: „Chronic obstructive pulmonary disease“
CRP: C‑reaktives Protein
DAIR: Debridement, Antibiotikatherapie und Implantaterhalt
EBJIS: European Bone and Joint Infection Society
PPI: Periprothetische Infektion
STAU: *Staphylococcus aureus*
